# Task geometry alignment enables parameter independent and accurate genomic search

**DOI:** 10.1038/s41598-026-65239-4

**Published:** 2026-08-05

**Authors:** Justin Boone

**Affiliations:** Fernuniversität Hagen, Hagen, Germany

**Keywords:** Computational biology and bioinformatics, Mathematics and computing

## Abstract

Standard sequence alignment models biological homology through the metric space of edit distance. While effective for global orthology, this rigid geometric assumption struggles with discrete biological realities–such as insertions/deletions (indels) and fragment-to-reference asymmetry–forcing a reliance on heuristic gap penalties. To address this, we propose Task-Geometry Alignment (TGA), a design principle that structurally aligns algorithmic representation with the intrinsic geometry of the biological task. We implement TGA in TGAlign, an expert-parameter-independent tool that tiles reference databases to match query lengths, encodes sequences into gap-robust syncmer profiles, and indexes them for high-speed Approximate Nearest Neighbor (ANN) search. Benchmarking against leading aligners (USEARCH, VSEARCH, MMseqs2) demonstrates performance strictly bounded by biological architecture. On standard substitution-heavy markers (COI), TGAlign achieves statistical parity with the state-of-the-art. Conversely, on sequence fragments and indel-heavy markers, TGAlign yields statistically significant accuracy improvements (up to 10% on 16S) while matching the peak performance of MMseqs2 on highly variable ITS datasets. By translating sequence comparison into dense matrix operations via ANN indexing, the current implementation maintains sub-millisecond query latency–an order-of-magnitude reduction over traditional aligners–providing a robust and scalable framework for post-alignment genomic search. Source code is available at https://github.com/JustinBooneLab/TGAlign and datasets are archived at Zenodo (DOI: 10.5281/zenodo.17973054).

## Introduction

The dominant paradigm for genomic identification—pairwise alignment—evaluates homology through the continuous metric space of edit operations^1,2^. While highly effective for continuous global orthology, this mathematical abstraction conflicts with the true geometry of biological sequence space, which is fundamentally discrete, distorted by insertions and deletions (indels), and characterized by severe length asymmetries (e.g., matching fragments to full-length genes)^3^. Faced with this *geometric mismatch*, practitioners must manually coerce algorithms via parameter tuning—such as adjusting heuristic gap penalties—to approximate the correct behavior. This reliance on runtime optimization introduces significant user-dependent variability, making outcomes highly contingent on configuration proficiency rather than the underlying biological data^4^.

In practice, this geometric mismatch manifests in two primary forms. First, evolutionary insertions and deletions (indels) create localized structural distortions. Traditional alignment attempts to span these via rigid gap penalties, which often fail when indels are massive or frequent. TGA counters this by utilizing gap-robust *k*-mer sketching^5,6^, which evaluates local sequence neighborhoods independently and bypasses the need for contiguous global alignment. Second, modern sequencing frequently generates extreme length asymmetry, such as mapping short environmental reads against full-length reference genes. Pairwise aligners inherently struggle to map a small geometry onto a large one without extensive heuristic tuning. TGA resolves this architecturally by tiling the large reference sequences into discrete, overlapping windows that match the length of the query, converting an asymmetric mapping problem into a balanced nearest-neighbor search.

**Fig. 1:**
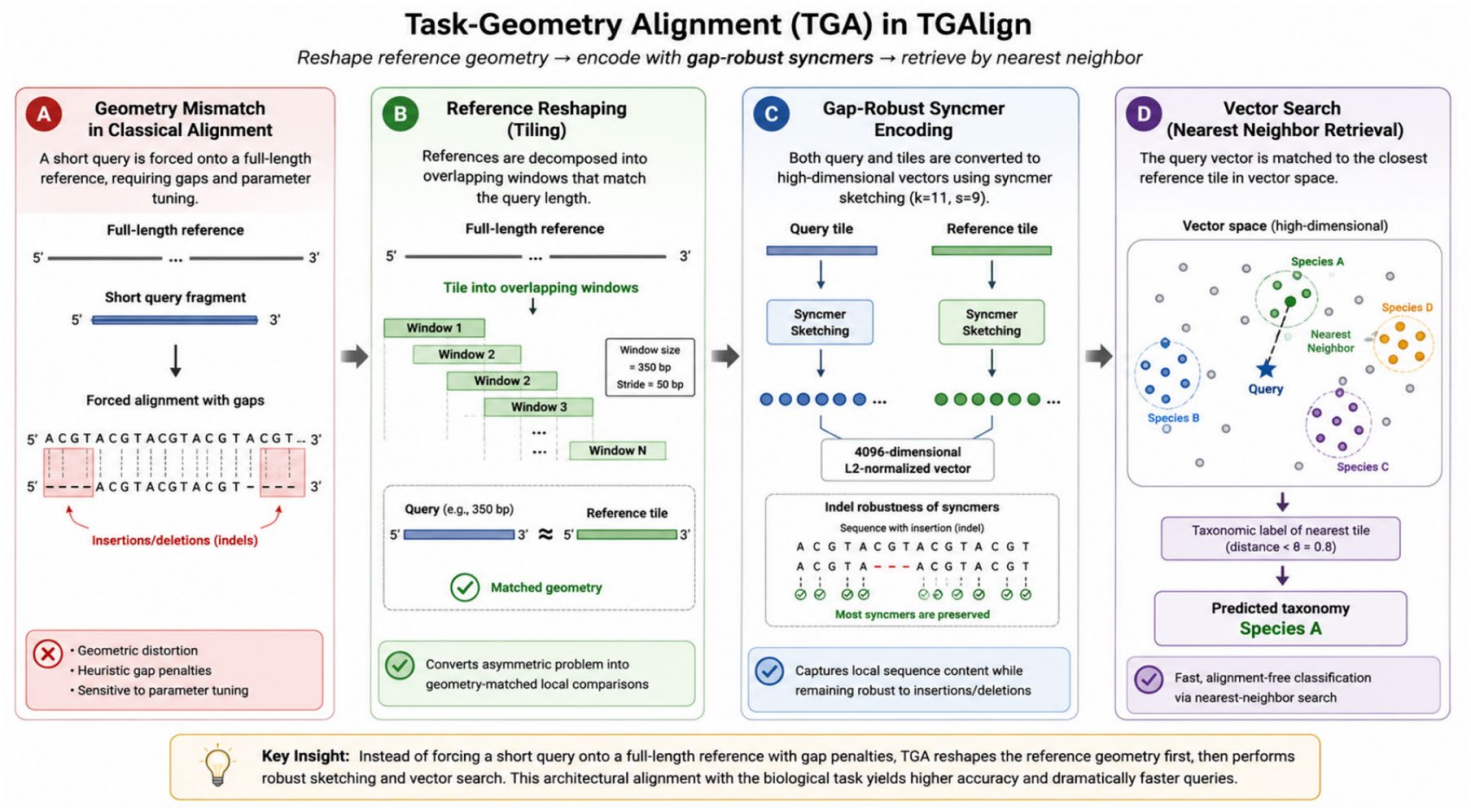
Schematic overview of task-geometry alignment (TGA) in TGAlign. (**A**) Classical alignment forces a rigid geometric comparison, struggling with asymmetric lengths and relying on heuristic gap penalties to handle indels. (**B**) TGA resolves this by architecturally reshaping the reference database into sliding windows congruent with the expected query length. (**C**) Gap-robust feature extraction is achieved via Syncmer sketching^7^, which preserves local sequence signatures even when insertions or deletions cause frameshifts. (**D**) Sequences are projected into a 4096-dimensional Euclidean space, transforming the complex alignment problem into an ultrafast Approximate Nearest Neighbor (ANN) vector search via library indexing^8^.

To resolve this, we propose Task-Geometry Alignment (TGA), a design principle predicated on structural congruence: shifting optimization from volatile user-tuned variables to fixed architectural layouts that mirror the intrinsic geometry of the biological task. TGA posits a three-step framework: (1) computationally reshaping the reference database (e.g., tiling) to match query geometry; (2) applying a gap-robust encoding, such as *k*-mer sketching, and specifically Syncmers^7^; and (3) projecting the resulting vectors into high-dimensional space for Approximate Nearest Neighbor (ANN) retrieval^8^. We instantiate this principle for DNA barcoding^9^ with TGAlign, a highly automated tool that validates the TGA hypothesis. Across five benchmarks encompassing leading state-of-the-art aligners, TGAlign achieves statistically superior accuracy (up to 10%) on indel-heavy data and an order-of-magnitude reduction in query latency over traditional heuristics. Crucially, by solving the geometric mismatch structurally through architecturally frozen constants rather than user-tuned variables, TGAlign autonomously matches or exceeds expert-configured layouts on fragment identification tasks. By decoupling biological homology from standard alignment pipelines, this work establishes TGA as a highly reproducible framework for post-alignment genomic software design (Fig. 1).

## Results

We evaluated the TGA architecture–instantiated in TGAlign–against a comprehensive suite of industry-standard tools: USEARCH^10^ (the established baseline), VSEARCH^11^ (its open-source equivalent), and MMseqs2^12^ (the current state-of-the-art for ultrafast clustering). Utilizing a rigorous 5-fold cross-validation protocol, the comparative benchmarks demonstrate that performance dynamics track the underlying biological variation of the evaluation datasets.

### Indel-dominated data: performance under structural frameshifts

Consistent with the premise that continuous alignment metrics encounter boundaries when mapping discrete biological frameshifts, TGAlign demonstrated its most notable advantages on markers characterized by high insertion/deletion (indel) rates (Table 1). On the 16S V4 benchmark, TGAlign achieved 86.15% accuracy, outperforming the highest-scoring baseline aligner (VSEARCH) by 10.56% (*p łe 0.0001*). On the highly variable fungal ITS dataset, TGAlign achieved 47.37% accuracy, establishing statistical parity with the peak accuracy of the exhaustive MMseqs2 configuration (47.72%, *p > 0.05*) while demonstrating a distinct advantage over standard heuristic aligners.

Concurrently, TGAlign executed these queries with sub-millisecond query latencies (0.09–0.41 ms). This represents a 4*×* to 6*×* runtime reduction over traditional sequential heuristic aligners, though it is important to note that MMseqs2 latencies in this benchmarking environment include disk I/O cross-validation overhead and do not reflect peak database throughput.

**Table 1:** Performance on indel-heavy datasets.

Dataset	Algorithm	Accuracy (%)	Macro F1 (%)	Query Time (ms)
16S V4	TGAlign	**86.15 ± 0.57**	**58.14 ± 3.11**	**0.09 ± 0.01**
	VSEARCH	75.59 ± 1.06	55.27 ± 3.72	0.59 ± 0.01
	USEARCH	75.09 ± 1.03	55.00 ± 3.70	0.33 ± 0.01
	MMseqs2	75.59 ± 1.56	54.48 ± 3.52	44.79*
ITS (Fungi)	TGAlign	47.37 ± 0.85	33.91 ± 1.09	**0.41 ± 0.03**
	MMseqs2	**47.72 ± 1.39**	**34.95 ± 1.81**	54.72*
	VSEARCH	44.32 ± 1.52	34.06 ± 2.05	7.78 ± 0.04
	USEARCH	43.08 ± 1.30	33.25 ± 1.97	1.74 ± 0.05

TGAlign demonstrates statistically significant accuracy gains on 16S targets while matching the state-of-the-art on complex ITS markers, executing queries at a fraction of the latency of traditional aligners.

MMseqs2 latencies (*) include validation framework disk I/O overhead.

### Substitution-dominated data: evaluation on continuous homology

To verify the baseline behavior of vector-space projection on sequences lacking severe geometric distortion, we evaluated performance on full-length COI barcodes and a taxonomically balanced variant. In these substitution-heavy scenarios, TGAlign achieved statistical parity with all three state-of-the-art alignment baselines (*p > 0.17*, Supplementary Table S2 and Supplementary Table S3). This indicates that the Euclidean comparison of syncmer profiles captures baseline biological homology with accuracy equivalent to global alignment while executing queries within a significantly reduced time envelope.

### Automated resolution of fragment asymmetry

Addressing the structural challenge of identifying short reads against full-length references, TGAlign resolved fragment asymmetry without requiring user-defined coverage or alignment constraints (Table 2). TGAlign achieved 72.67% accuracy, significantly exceeding the expert-configured VSEARCH (71.41%) and USEARCH (70.92%) fragment-optimized baselines (*p łe 0.00035*).

To isolate the specific mechanism driving this performance, we performed an ablation study disabling the reference tiling protocol. This architectural removal resulted in a $\sim 9.2\%$ accuracy collapse (Supplementary Table S1), statistically confirming that resolving structural length asymmetry via geometric reference reshaping prior to feature extraction is the primary driver of robust fragment identification.

**Table 2:** Performance on sequence fragments (COI).

Method	Accuracy (%)	Macro F1 (%)	Query time (ms)
TGAlign (Parameter-Independent)	**72.67 ± 1.08**	**60.96 ± 1.68**	**0.85 ± 0.05**
MMseqs2	71.50 ± 1.15	60.64 ± 1.57	43.64*
VSEARCH (Fragment Expert)	71.41 ± 1.20	60.49 ± 1.67	3.19 ± 0.05
USEARCH (Fragment Expert)	70.92 ± 1.19	60.13 ± 1.92	1.40 ± 0.02
USEARCH (Standard/Naive)	70.99 ± 1.21	60.19 ± 1.93	1.15 ± 0.01

By structurally resolving geometric asymmetry via database tiling, the parameter-independent TGAlign significantly outperforms both standard and expert-configured alignment heuristics.

## Discussion

While TGAlign establishes a strong performance baseline for DNA barcoding, its primary contribution is the empirical validation of Task-Geometry Alignment (TGA). This design philosophy reframes bioinformatic software development: by encoding structural invariants directly into fixed architectural layouts–rather than exposing them as volatile, user-tuned runtime parameters–the system achieves high accuracy on complex biological geometries while remaining highly computationally efficient.

### Fixed architecture vs. volatile parameterization

The central implication of the TGA framework is that structural congruence can mitigate the necessity for runtime parameter optimization. Historically, user-driven parameter tuning has introduced significant variability across computational pipelines. The ablation results (Section 2.3) demonstrate that removing the geometrically congruent reference tiling step triggers a significant drop in fragment identification accuracy, confirming that structural alignment, rather than parameter tuning, serves as the primary determinant of success. By embedding empirically optimized constants directly into the architecture (Section 4.2), the system shifts the locus of optimization from user-tuned runtime variables to an expert-parameter-independent framework, ensuring strict reproducibility across research and diagnostic applications.

### Latency mechanics and computational scalability

To evaluate the mechanisms driving TGAlign’s speed advantage, we profiled the query latency into its constituent components (Supplementary Section S1.6). This micro-benchmarking revealed that feature extraction (C++ syncmer hashing) requires less than 0.15 ms per sequence. The remainder of the sub-millisecond query time is consumed by the Approximate Nearest Neighbor (ANN) search via FAISS. This highlights why TGA circumvents the dynamic programming bottlenecks of traditional alignment: by front-loading the computational effort into a one-time reference indexing step, the sequence matching process is transformed into highly optimized, sub-linear matrix operations.

While current single-threaded CPU benchmarks demonstrate a *2×* to *6×* reduction in query latency against optimized heuristic aligners, this serves as a conservative lower bound for the throughput potential of the architecture. Because vector search reduces natively to dense matrix operations, this framework can leverage SIMD acceleration and GPU parallelization to scale efficiently. Notably, TGAlign requires a higher initial computational investment to build the FAISS index (approximately 5–12 seconds across the baseline datasets) than traditional aligners. However, in production environments where reference databases remain largely static and are queried iteratively, this initial linear build cost is rapidly amortized by the sub-millisecond retrieval speeds.

### Applicable scope and operational limitations

To objectively define the boundaries of the current TGAlign implementation, we outline its operational scope and structural limitations. The tool is presently optimized for nucleotide sequences and specifically validated on standard, indel-prone barcode markers (e.g., COI, 16S, ITS). Consequently, the software in its current form cannot be applied directly to amino acid sequence classification, as protein space requires distinct, substitution-matrix-aware sketching paradigms.

Furthermore, while the TGA principle is theoretically extensible to long-read sequencing technologies, the current syncmer parameters have not been calibrated for their distinct error profiles. Finally, from a deployment perspective, the current implementation utilizes an Inverted File Index (IndexIVFFlat) for ANN retrieval. While setting nprobe = 5 virtually eliminates quantization error at the current scale, this index still stores full uncompressed vectors in RAM. Scaling to billion-level database scales will necessitate transitioning to compressed hierarchical indexing topologies (e.g., HNSW or IVF-PQ), representing an immediate next step in development.

### A structural roadmap for post-alignment genomics

The utility of the TGA principle extends beyond barcode identification. Its core protocol–tiling references to match query geometry, applying gap-robust sketches, and performing vector-space retrieval–provides a reproducible framework for addressing sequence-matching tasks constrained by traditional alignment bottlenecks. Future applications include viral quasispecies analysis via targeted genome tiling, alignment-free long-read error correction via self-tiling, and metagenomic contig classification. By prioritizing the structural relationship between query and reference geometries, TGA offers a pathway toward evaluating biological homology through geometric alignment rather than base-level identity.

## Methods

### Datasets

All datasets are permanently archived on Zenodo (DOI: 10.5281/zenodo.17973054). We curated three primary datasets: COI Full-Length, comprising 600–700 bp animal sequences from NCBI corresponding to the standard barcode region^9^, and ITS (Fungi), consisting of fungal ITS sequences from NCBI^13^. The 16S V4 dataset was extracted *in silico* from the Greengenes 13.8 database^14^ using primers 515F/806R^15^. To evaluate partial matching, we generated the COI Fragments test set by extracting random 300 bp substrings from held-out references, excluding any sequences with ambiguous bases (‘N’). Finally, to control for taxonomic bias, we constructed the COI (Balanced-10) dataset by downsampling the full COI set to a maximum of 10 sequences per genus, following the dataset balancing protocol established by Edgar^16^.

### TGAlign TGA implementation

TGAlign instantiates the TGA principle via a three-stage pipeline. To ensure reproducibility, the framework shifts the locus of optimization from user-tuned runtime variables to a set of fixed, architecturally frozen constants. First, to enforce structural matching with query sequences, reference sequences *>350* bp are decomposed into overlapping windows using a 350 bp window and a 50 bp stride, with remaining trailing sequences shorter than the window size omitted. The selection of these dimensions was driven by biological intuition: a 350 bp window approximates the typical length of environmental barcode fragments, while the 50 bp stride provides dense overlapping coverage, ensuring that any arbitrary query fragment structurally aligns with at least one reference window with minimal edge truncation.

Second, each sequence or window is projected into a 4096-dimensional $L_2$-normalized vector using syncmer sketching (*k=11*, *s=9*)^7^. The core sketching engine is implemented in C++ with a pybind11 interface^17^. Specifically, the algorithm computes canonical *k*-mers using a strand-symmetric selection rule (accepting *k*-mers where the minimal *s*-mer occurs at offset 0 or *k-s*). Selected *k*-mers are hashed using a 64-bit integer mix function, and the resulting hash modulo 4096 determines the index of the feature vector to be incremented. This dimensionality (4096) was selected as it provides an optimized memory trade-off, minimizing hash collision rates while natively supporting SIMD-accelerated $L_2$ distance calculations.

Third, the resulting vectors are indexed for high-speed similarity search using the FAISS IndexIVFFlat library^8^, utilizing a probe parameter (nprobe=5) to virtually eliminate quantization error and approximate exact Euclidean retrieval. Queries are assigned the taxonomic label of their nearest neighbor only if the squared $L_2$ distance falls below the threshold *θ = 0.8*, which was selected empirically during pilot cross-validation iterations to optimize the precision-recall boundary.

Crucially, Stage 1 (Reference Reshaping) represents the primary architectural contribution of the TGA design principle. By physically decomposing the reference library into balanced target geometries, we systematically eliminate structural length asymmetry before any sequence comparison occurs. Stages 2 and 3 subsequently leverage these balanced vector configurations to execute downstream searches at sub-linear speeds, as formalized below in Algorithm 1.

**Algorithm 1:**
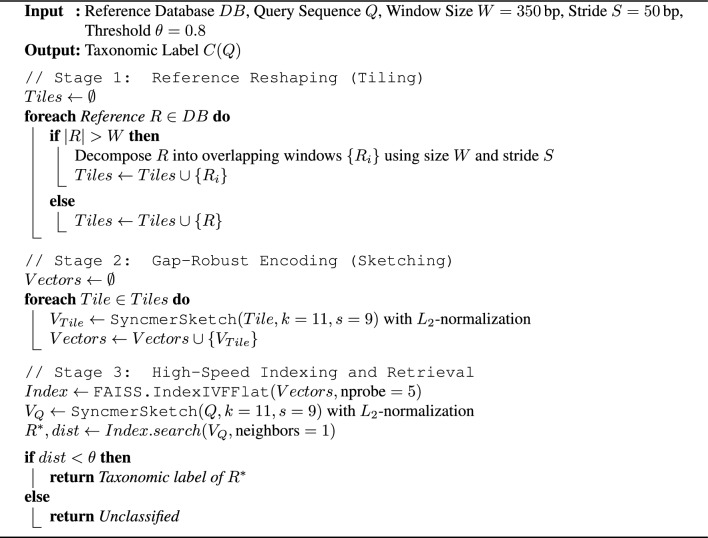
TGAlign task-geometry alignment pipeline.

### Benchmarking and statistics

To evaluate local sequence classification performance, we benchmarked TGAlign against three industry-standard tools widely used for high-confidence taxonomic assignment: USEARCH v12.0^10^, VSEARCH v2.28.1^11^, and MMseqs2^12^. Benchmarks were executed using a 5-fold stratified cross-validation scheme ($\text {seed}=42$) implemented in scikit-learn^18^.

To ensure a rigorous baseline, USEARCH and VSEARCH were executed in global alignment mode with production parameters:-strand both to account for reverse-complement orientation, and-maxaccepts 1 -maxrejects 32 for standard heuristic acceleration. MMseqs2 was evaluated at maximum sensitivity (-s 7) with nucleotide search mode enabled. Identity thresholds were set to 0.97 for COI/16S and 0.985 for ITS. For the “Expert” fragment configuration, we additionally enforced a query coverage constraint (-query_cov 0.9) on the alignment tools to filter partial matches.

To accurately profile native matrix operations via FAISS, hardware resources were normalized to a single thread, and all benchmarks were executed locally on an Apple M-series (ARM64) architecture leveraging native BLAS optimization.

Performance was quantified using classification accuracy and Macro-Averaged F1-Score to ensure robustness against class imbalance. Statistical significance of performance differences was assessed using two-tailed paired t-tests (*p < 0.05*) on per-fold scores.

## Supplementary Information

Supplementary Information.

## Data Availability

The datasets generated and analysed during the current study are available in the Zenodo repository, [https://doi.org/10.5281/zenodo.17973054]. The source code for the TGAlign algorithm and benchmarking scripts is available on GitHub at [https://github.com/JustinBooneLab/TGAlign].

## References

[1] Altschul, S. F., Gish, W., Miller, W., Myers, E. W. & Lipman, D. J. Basic local alignment search tool. *J. Mole. Biol.* **215**, 403–410 https://www.sciencedirect.com/science/article/pii/S0022283605803602. (1990). 10.1016/S0022-2836(05)80360-2 2231712

[2] Zielezinski, A., Vinga, S., Almeida, J. & Karlowski, W. Alignment-free sequence comparison: Benefits, applications, and tools. *Genome Biol.* **18**, 186 (2017). 28974235 10.1186/s13059-017-1319-7 PMC5627421

[3] Vinga, S. & Almeida, J. Alignment-free sequence comparison - a review. *Bioinformatics (Oxford, England)* **19**, 513–23 (2003). 12611807 10.1093/bioinformatics/btg005

[4] Kumar, S., Singh, M., Sharma, R. & Gupta, M. K. Chapter 4 - systematic benchmarking of omics computational tools. In Gupta, M. K., Katara, P., Mondal, S. & Singh, R. L. (eds.) *Integrative Omics*, 55–83 (Academic Press, 2024). https://www.sciencedirect.com/science/article/pii/B9780443160929000047.

[5] Ondov, B. Mash: Fast genome and metagenome distance estimation using minhash. *Genome Biol.* 17, (2016). 10.1186/s13059-016-0997-x PMC4915045 27323842

[6] Li, H. Minimap2: Pairwise alignment for nucleotide sequences. *Bioinformatics (Oxford, England)* **34** (2018). 10.1093/bioinformatics/bty191 PMC6137996 29750242

[7] Edgar, R. Syncmers are more sensitive than minimizers for selecting conserved k-mers in biological sequences. *PeerJ* **9**, e10805 (2021). 33604186 10.7717/peerj.10805 PMC7869670

[8] Douze, M. et al. The faiss library. *IEEE Transactions on Big Data* **12**, 346–361 (2026).

[9] Hebert, P., Cywinska, A., Ball, S. & deWaard, J. Biological identification through dna barcodes. *Proc. R. Soc. London B* **270**, 313–321 (2003). 10.1098/rspb.2002.2218 PMC1691236 12614582

[10] Edgar, R. Search and clustering orders of magnitude faster than blast. *Bioinformatics (Oxford, England)* **26**, 2460–1 (2010). 20709691 10.1093/bioinformatics/btq461

[11] Rognes, T., Flouri, T., Nichols, B., Quince, C. & Mahé, F. Vsearch: a versatile open source tool for metagenomics. *PeerJ* **4** (2016). 10.7717/peerj.2584 PMC5075697 27781170

[12] Steinegger, M. & Söding, J. Mmseqs2 enables sensitive protein sequence searching for the analysis of massive data sets. *Nature Biotechnol.* **35** (2017). 10.1038/nbt.3988 29035372

[13] Schoch, C. Nuclear ribosomal internal transcribed spacer (its) region as a universal dna barcode marker for fungi. proc natl acad sci usa. *Proc. Natl. Acad. Sci.* 24, (2012). 10.1073/pnas.1117018109 PMC3341068 22454494

[14] DeSantis, T. et al. Greengenes, a chimera-checked 16s rrna gene database and workbench compatible with arb. *Appl. Environ. Microbiol. - AEM* **72**, 5069–5072 (2006). 10.1128/AEM.03006-05 PMC1489311 16820507

[15] Caporaso, J. et al. Global patterns of 16s rrna diversity at a depth of millions of sequences per sample. *Proceedings of the National Academy of Sciences of the United States of America* **108**(Suppl 1), 4516–22 (2011). 20534432 10.1073/pnas.1000080107 PMC3063599

[16] Edgar, R. C. Accuracy of taxonomy prediction for 16s rrna and fungal its sequences. *PeerJ* **6**, e4652 (2018). 29682424 10.7717/peerj.4652 PMC5910792

[17] Wenzel, J. pybind11 – seamless operability between c++11 and python. https://github.com/pybind/pybind11 (2017).

[18] Pedregosa, F. Scikit-learn: Machine learning in python. *J. Mach. Learn. Res.* 12, (2012).

