# Supplementary material for: Task geometry alignment enables parameter independent and accurate genomic search
Source: Sci Rep. 2026 Aug 5;16:24173. doi: 10.1038/s41598-026-65239-4 (PMC13442880; doi:10.1038/s41598-026-65239-4)
Supplement: Supplementary file 1 [file 41598_2026_65239_MOESM1_ESM.pdf]

Supplementary Information: Task-Geometry Alignment provides a design principle for parameter-free and accurate genomic search

Justin Boone[1,*]
[1]Fernuniversität Hagen, Hagen, Germany
[*]Corresponding author: justin.boone@studium.fernuni-hagen.de

S1 Supplementary Information

S1.1 Data Sourcing and Generation

To ensure full reproducibility, we provide the exact query parameters used to generate the primary datasets. All resulting FASTA files are permanently archived on Zenodo.

S1.1.1 NCBI Datasets (COI and ITS)

Datasets were generated via Entrez queries against the NCBI Nucleotide database (accessed 16.12.2025).

- **COI (Full-Length & Fragments):**
  - Query: `"cytochrome c oxidase subunit I"[All Fields] AND "Animalia"[Organism] AND 500:800[Sequence Length]`
  - Limit: 10,000 records
- **ITS (Fungi):**
  - Query: `"internal transcribed spacer"[All Fields] AND "Fungi"[Organism] AND 200:1000[Sequence Length]`
  - Limit: 5,000 records

S1.1.2 Greengenes Dataset (16S V4)

Extracted from Greengenes v13.8 (accessed 16.12.2025).

- Source: `gg_13_8_otus/rep_set/99_otus.fasta`
- Taxonomy: `gg_13_8_otus/taxonomy/99_otu_taxonomy.txt`

S1.2 Ablation Study: Validating the Architectural Heuristic

To isolate the causal contribution of the Task-Geometry Alignment (TGA) principle, we conducted an ablation study disabling the sliding-window heuristic. This forced a "naive" comparison between short query sketches and full-length reference sketches.

To ensure the statistical validity of the ablation comparison, the dataset was filtered to include only taxa with ≥ 5 sequences. Out of 6,407 raw sequences, 6,268 remained after N-filtering, resulting in a final parsed dataset size of 3,838 sequences. The results of the 5-fold cross-validation replication (Table S1) reveal a highly significant accuracy drop when TGA is disabled. This confirms that the tiling architecture—and not the syncmer sketching algorithm alone—is the fundamental driver of TGALIGN's robustness in fragment identification.

Table S1: Impact of TGA ablation on fragment classification. Performance metrics compare the standard TGALIGN (TGA active) against the ablated version (TGA disabled) across a 5-fold cross-validation split. The significant average degradation of 9.22% confirms the necessity of the sliding-window heuristic for resolving geometric mismatch.

Benchmark Dataset Average	Configuration	Fold 1	Fold 2	Fold 3	Fold 4	Fold 5
COI Fragments **91.79%**	Standard (TGA)	91.67%	92.32%	92.19%	90.61%	92.18%
(Filtered ≥ 5) **82.57%**	Ablated (Naive)	83.98%	82.81%	82.03%	80.70%	83.31%
**Performance Drop** $-\mathbf{9.22}\%$		-7.69%	-9.51%	-10.16%	-9.91%	-8.87%

Note on Indexing Dynamics: During the indexing phase of this sub-sampled ablation study, training FAISS on 3,070 vector points per fold to cluster into a static 200 centroids would typically yield an under-saturated centroid allocation warning. To handle this dynamically without user intervention, `TGAlign` implements an automated cluster scaling heuristic:

$$\text{nlist} = \max(1, \min(N \times 5//40, 200))$$

scaling down the Voronoi partitioning centroids for small reference architectures to ensure a stable indexing layout configuration.

S1.3 Comprehensive Benchmarking Results

Table S2 details the complete performance metrics for all experimental conditions, including computational costs. While TGAlign incurs a higher one-time index construction cost compared to USEARCH (due to the generation of tiled syncmer sketches), it achieves significantly faster query speeds (Avg. Query Time) across all datasets. P-values represent the statistical significance of the accuracy difference between TGAlign and the primary USEARCH baseline (Global alignment).

Table S2: Comprehensive Performance Metrics (TGAlign vs. USEARCH Baseline under Mixed Orientation). Detailed breakdown of 5-fold cross-validation results with a 50% reverse-strand query injection. For the COI Fragments task, comparison is made against the USEARCH "Expert" configuration. P-values denote the statistical significance of the accuracy difference; 'ns' indicates no significant difference ($p > 0.05$).

Dataset	Algorithm	Accuracy (%)	Macro F1 (%)	Build Time (s)	Query Time (ms)	p-value
16S V4	TGALIGN	86.15 ± 0.57	58.14 ± 3.11	0.2362 ± 0.0272	0.0893 ± 0.0080	0.00003
	USEARCH	75.09 ± 1.03	55.00 ± 3.70	0.0015 ± 0.0001	0.3316 ± 0.0092	
ITS (Fungi)	TGALIGN	47.37 ± 0.85	33.91 ± 1.09	1.9626 ± 0.0116	0.4088 ± 0.0261	0.00037
	USEARCH	43.08 ± 1.30	33.25 ± 1.97	0.0036 ± 0.0001	1.7373 ± 0.0469	
COI Full	TGALIGN	71.23 ± 1.22	60.66 ± 1.46	2.4728 ± 0.0063	0.9366 ± 0.0750	1.00000 (ns)
	USEARCH	71.23 ± 1.30	60.78 ± 1.59	0.0041 ± 0.0005	2.4557 ± 0.0048	
COI Bal-10	TGALIGN	59.21 ± 1.66	55.49 ± 1.67	1.4564 ± 0.0444	0.6790 ± 0.0594	0.07048 (ns)
	USEARCH	59.09 ± 1.60	55.43 ± 1.63	0.0035 ± 0.0022	2.8889 ± 0.8253	
COI Frag	TGALIGN	72.67 ± 1.08	60.96 ± 1.68	2.4966 ± 0.0754	0.8457 ± 0.0505	0.00025
	USEARCH (Exp)	70.92 ± 1.19	60.13 ± 1.92	0.0036 ± 0.0001	1.4037 ± 0.0158	

S1.4 Hyperparameter Optimization

S1.4.1 Distance Threshold Selection

We performed a threshold sweep on the COI Full-Length dataset to balance classification accuracy against the sequence rejection rate. As shown in Figure S1, the classification accuracy remains consistently high, hovering between 95.5% and 97.5% across the entire range of evaluated thresholds. Concurrently, a distinct "elbow" in the rejection rate curve occurs between 0.6 and 0.7, whereafter the curve flattens. We selected a distance threshold of 0.8 as our baseline operating point; at this threshold, TGALIGN operates on a stable plateau of high fidelity ($\sim 96.7\%$ accuracy) while maintaining a conservative rejection rate ($\sim 17\%$), successfully optimizing the yield of confident taxonomic assignments.

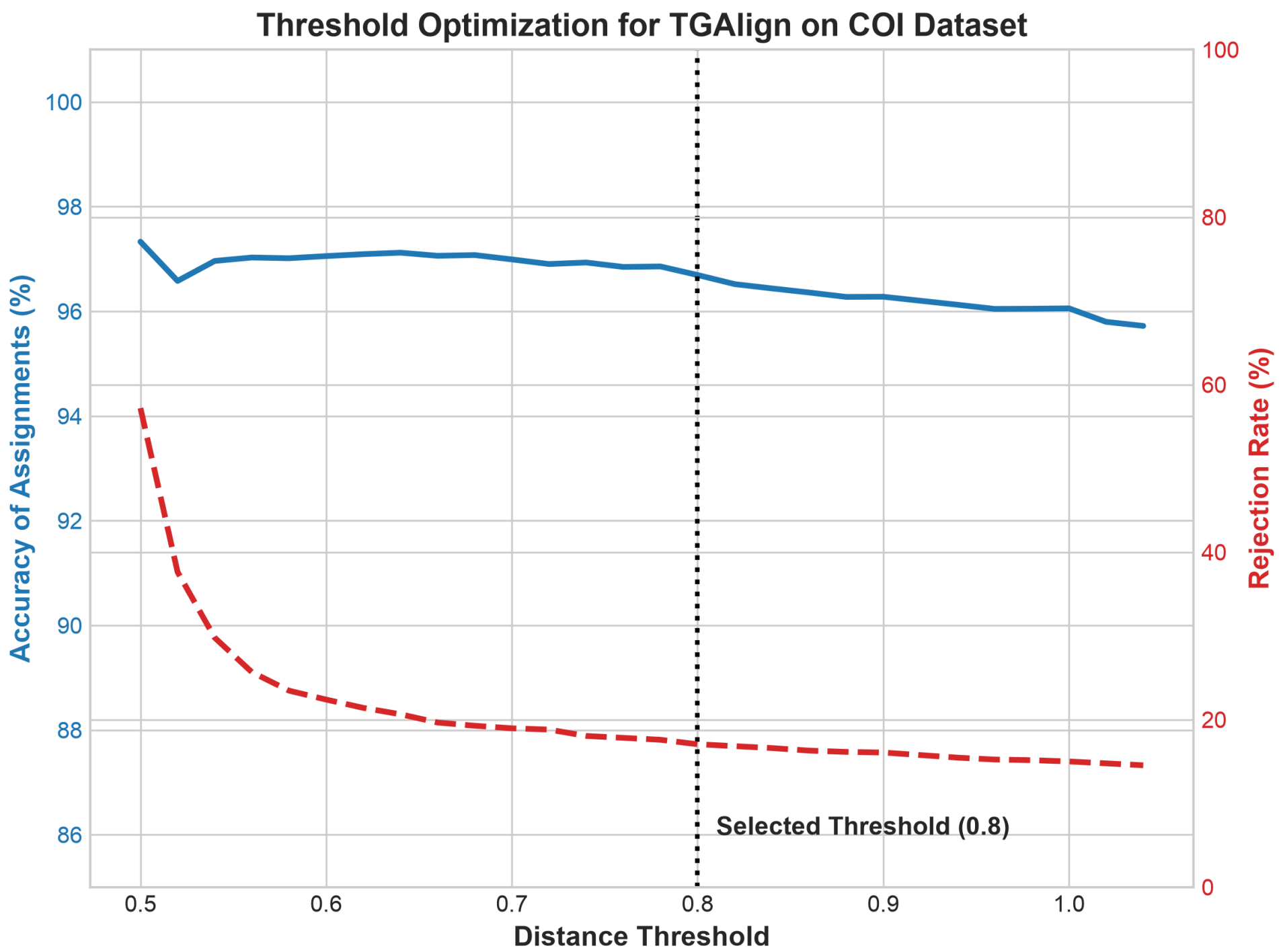

Figure S1: Accuracy vs. Rejection Rate trade-off across distance thresholds. The plot tracks taxonomic assignment accuracy (solid blue curve, left axis) and the percentage of rejected (unclassified) sequences (dashed red curve, right axis). The selected threshold of 0.8 (vertical dotted line) achieves a highly favorable trade-off, securing $\sim 96.7\%$ accuracy while keeping the sequence rejection rate restricted to $\sim 17\%$.

S1.4.2 Syncmer k-mer Size Selection

We optimized the syncmer k-mer size k (with $s = k - 2$) via 5-fold cross-validation on the COI Full-Length dataset. Figure S2 illustrates the performance trajectory across the k = 9–15 range. Mean classification accuracy peaks at $k = 9$ ($\sim 81.5\%$) and declines gradually to $\sim 80.0\%$ at $k = 15$. Although $k = 9$ yields marginally higher raw accuracy, the performance differences across the high-accuracy k = 9–11 plateau are statistically minor (with heavily overlapping standard deviation error bars). We selected $k = 11$ (yielding $\sim 81.1\%$ accuracy) as the default architecture setting to prioritize biological specificity and reduce the risk of false-positive matches arising from short, spurious k-mer hits.

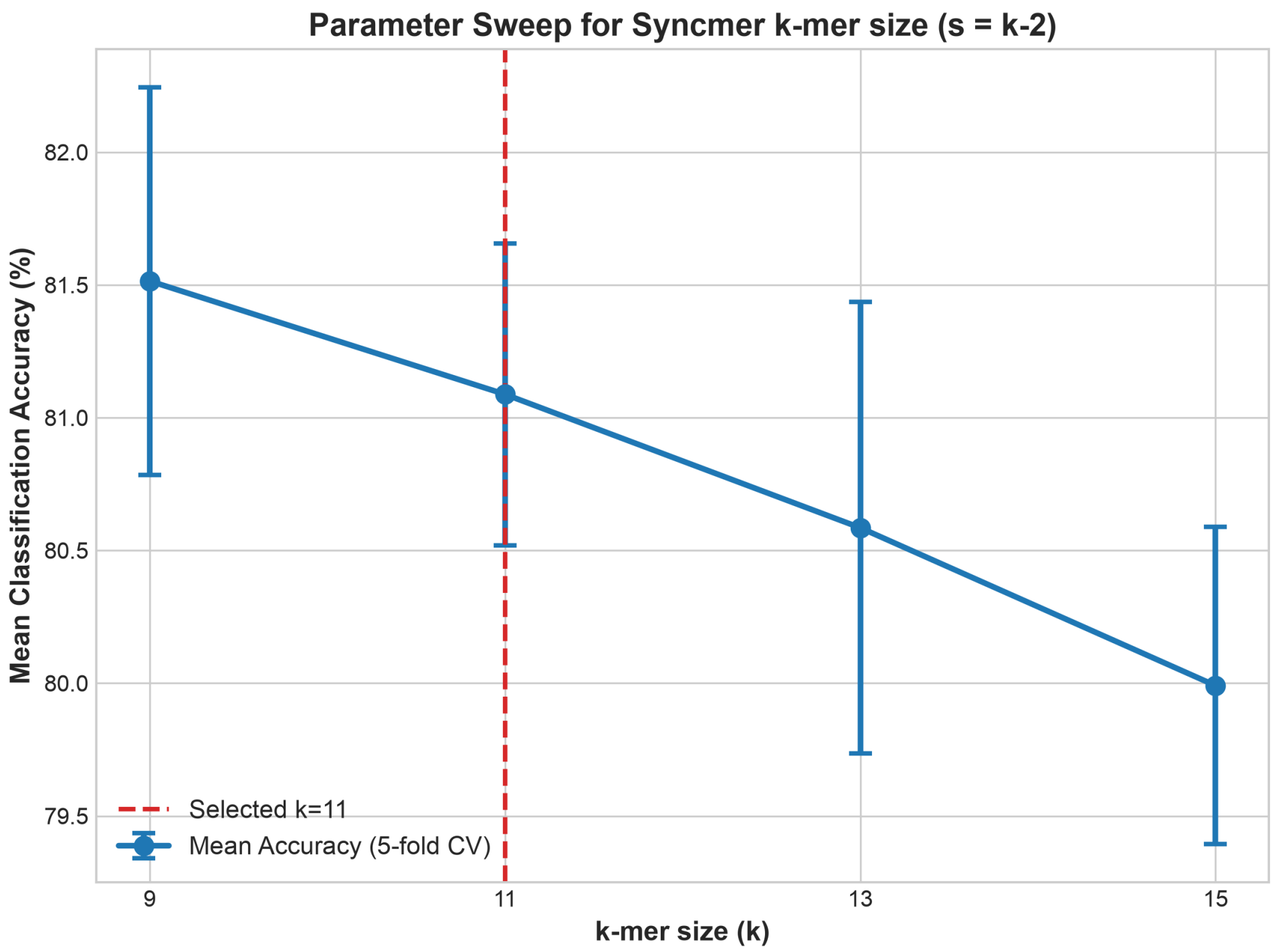

Figure S2: Parameter sweep for Syncmer k-mer size. Mean classification accuracy ($\pm$ SD) is tracked across a range of k values ($s = k - 2$) using 5-fold cross-validation. The selected parameter of $k = 11$ (dashed red line) sits securely within the optimal performance plateau ($k = 9$–11) while inherently favoring greater sequence specificity.

S1.5 Comparison with State-of-the-Art (VSEARCH and MMseqs2)

To ensure our validation reflects broader trends among alignment-based tools, we conducted an additional benchmark against VSEARCH (the open-source standard for amplicon analysis) and MMseqs2, the current state-of-the-art for high-throughput sequence search. Both tools were evaluated at maximum sensitivity with a single thread to match the baseline conditions.

The results (**Table** S3) show that MMseqs2 generally achieves comparable or slightly higher accuracy than the traditional VSEARCH aligner. Against this elevated baseline, TGAlign maintains a highly significant accuracy advantage on the 16S V4 dataset (+10.56% over both VSEARCH and MMseqs2), reinforcing the hypothesis that vectorization provides unique robustness to specific indel-heavy geometries. On the remaining substitution-heavy datasets, TGAlign operates at near-parity (within $\sim$1%) of the highest-scoring baselines, while consistently delivering the fastest query speeds across all conditions.

Note on Latency Metrics: The query times reported for MMseqs2 in this benchmark are highly artifactual. Because our cross-validation framework evaluates small batches ($\sim$500 sequences per fold), the wall-clock time for MMseqs2 is completely dominated by disk I/O and subprocess initialization (database creation and format conversion). Consequently, these timing metrics do not reflect the true algorithmic speed or throughput capabilities of MMseqs2 on large-scale datasets.

Table S3: Performance Comparison Between TGAlign, VSEARCH, and MMseqs2 on Mixed-Orientation Data. TGALIGN retains a robust accuracy edge on the indel-heavy 16S dataset, matches or outperforms the baselines on ITS and fragmented COI markers, and consistently registers sub-millisecond query latencies across all benchmarks.

Dataset	Algorithm	Accuracy (%)	Macro F1 (%)	Build Time (s)	Query Time (ms)
16S V4	TGALIGN	86.15 ± 0.57	58.14 ± 3.11	0.2362 ± 0.0272	0.0893 ± 0.0080
	VSEARCH	75.59 ± 1.06	55.27 ± 3.72	0.0014 ± 0.0002	0.5936 ± 0.0135
	MMseqs2	75.59 ± 1.56	54.48 ± 3.52	0.0512 ± 0.0092	44.7946 ± 2.8370
ITS (Fungi)	TGALIGN	47.37 ± 0.85	33.91 ± 1.09	1.9626 ± 0.0116	0.4088 ± 0.0261
	VSEARCH	44.32 ± 1.52	34.06 ± 2.05	0.0036 ± 0.0001	7.7757 ± 0.0409
	MMseqs2	47.72 ± 1.39	34.95 ± 1.81	0.0609 ± 0.0010	54.7162 ± 0.5725
COI Full	TGALIGN	71.23 ± 1.22	60.66 ± 1.46	2.4728 ± 0.0063	0.9366 ± 0.0750
	VSEARCH	71.28 ± 1.32	60.83 ± 1.60	0.0038 ± 0.0002	6.6860 ± 0.0505
	MMseqs2	71.21 ± 1.20	60.70 ± 1.45	0.0607 ± 0.0006	60.3419 ± 0.5818
COI Frag	TGALIGN	72.67 ± 1.08	60.96 ± 1.68	2.4966 ± 0.0754	0.8457 ± 0.0505
	VSEARCH	71.41 ± 1.20	60.49 ± 1.67	0.0038 ± 0.0003	3.1900 ± 0.0520
	MMseqs2	71.50 ± 1.15	60.64 ± 1.57	0.0618 ± 0.0029	43.6395 ± 2.5418
COI Bal-10	TGALIGN	59.21 ± 1.66	55.49 ± 1.67	1.4564 ± 0.0444	0.6790 ± 0.0594
	VSEARCH	59.09 ± 1.66	55.45 ± 1.73	0.0024 ± 0.0003	7.1474 ± 0.2297
	MMseqs2	59.13 ± 1.71	55.46 ± 1.71	0.0566 ± 0.0058	84.6539 ± 7.0830

S1.6 Latency Component Profiling

To address the mechanisms behind TGALIGN's speed advantage over traditional alignment, we performed a micro-benchmarking analysis to isolate the latency contributions of the two primary algorithmic components: Feature Extraction (C++ Syncmer Hashing) and Vector Search (FAISS Index querying). The profile was conducted on 1,000 synthetic COI-length (650 bp) sequences queried against a 5,000-sequence reference index.

As detailed in Table S4, the query latency is primarily comprised of the approximate nearest neighbor search via the FAISS IVFFlat index (75.5%), while the initial feature extraction phase compressing the DNA string into a 4096-dimensional vector is highly efficient, accounting for 24.5% of the total time. This validates the fundamental speed hypothesis of TGA: by front-loading the computational effort into a one-time vectorization step, the historically expensive $O(N)$ sequence comparison process is reduced to highly optimized, sub-linear matrix operations that outperform traditional alignment.

Table S4: Component Breakdown of Query Latency. Average processing time per 650 bp query sequence on a single CPU thread. Values are based on 1,000 queries against a 5,000-sequence reference index.

Component	Average Time (ms)	Relative Contribution (%)
Feature Extraction (C++ Syncmer Hashing)	0.1385	24.5%
Vector Search (FAISS IVFFlat, nprobe=5)	0.4263	75.5%
**Total Query Latency**	**0.5648**	**100.0%**
